# The effect of mangiferin on skin: Penetration, permeation and inhibition of ECM enzymes

**DOI:** 10.1371/journal.pone.0181542

**Published:** 2017-07-27

**Authors:** Renata Ochocka, Anna Hering, Justyna Stefanowicz–Hajduk, Krzysztof Cal, Helena Barańska

**Affiliations:** 1 Department of Biology and Pharmaceutical Botany, Medical University of Gdansk, Gdansk, Poland; 2 Department of Pharmaceutical Technology, Medical University of Gdansk, Gdansk, Poland; Helsingin Yliopisto, FINLAND

## Abstract

Mangiferin (2-C-β-D-glucopyranosyl-1,3,6,7-tetrahydroxyxanthone) is a polyphenol with strong antioxidant properties. Mangiferin is obtained from the mango tree (*Mangifera indica* L., *Anacardiaceae*). It has been proven that mangiferin exhibits many pharmacological activities. The aim of this study was to analyze the penetration of mangiferin into the human skin and through the skin. According to our knowledge, skin penetration and permeation studies of mangiferin have not been analyzed so far. Additionally, the influence of mangiferin on two Extracellular Matrix Enzymes (ECM): collagenase and elastase, was evaluated for the first time. It has been indicated that mangiferin is able to permeate the stratum corneum and penetrate into the epidermis and dermis in comparable amounts. For confirmation of the obtained results, fluorescence microscopy was successfully utilized. The analysis revealed the capability of mangiferin to reversibly inhibit elastase and collagenase activity. The mechanism of mangiferin interaction with both enzymes was estimated as a noncompetitive inhibition.

## Introduction

The skin is the first barrier and the biggest organ in the human body. During the natural process of ageing, the skin is the most sensitive barrier due to the influence of internal as well as external ageing. Natural (e.g. stratum corneum, melanin) and cosmetic barriers are not sufficient to protect living skin layers. The UV radiation passes through the skin and generates oxidative stress and free radicals. As a result, many ECM (Extracellular Matrix Enzymes) responsible for the degradation of skin macromolecules are activated [1,2]. This reduces the skin barrier and leads to skin dysfunction. As the consequence, the skin ageing process takes place more rapidly [3].

Collagen and elastin are extracellular proteins responsible for the specific structure and physical properties of the skin. Skin atrophy and flattening are caused mostly by the deterioration, reorganization and amorphisation of collagen and elastin fibers. Collagen confers strength and support to human skin, while elastin provides the elasticity of connective tissues [3]. In the skin macromolecules form specific structures that are extremely sensitive to UV radiation exposure. In the photoaged skin, elastin material is degraded by the elastase enzyme. This condition is called elastosis and is irreversible, thus the process of elastin synthesis is limited to the embryo formation [4]. Collagen fibers, on the other hand are synthesized throughout an individual’s lifetime, but in the elderly the process is not as intensive as in the young [5]. Both types of macroproteins: collagen and elastin, are degraded by matrix metalloproteinase enzymes (MMPs).

**Fig 1:**
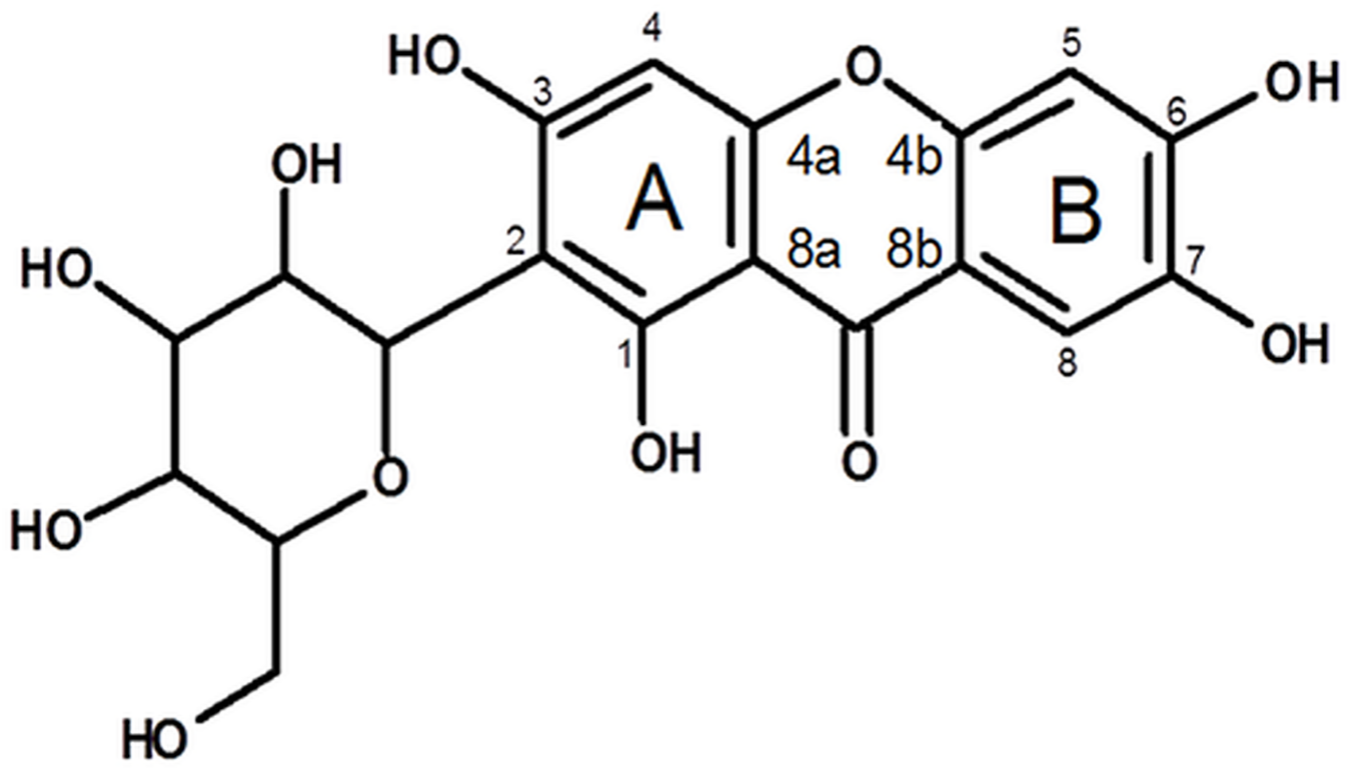
Mangiferin (C_19_H_18_O_11_).

There are many chemicals used to fight the free oxygen species generated by internal and external factors. Among them are many photoprotectors with different mechanisms of action, mainly on the skin’s surface [6]. A way to slow down the process of skin ageing is to use chemical or natural substances that should be able not only to ensure radioprotection but also to penetrate to the dermis and to inhibit the activation and/or activity of ECM enzymes. In recent years the interest in naturally occurring, less toxic and pharmacologically active compounds has increased. Molecules obtained from plants are used as a source of protective agents against oxidative stress [7]. A candidate to be used in such activity is mangiferin, the C-glucosylxanthone widely distributed in plants of tropical and subtropical regions (Fig 1). Mangiferin is obtained from the mango tree (*Mangifera indica*, L. *Anacardiaceae*), although a significant amount of mangiferin can also be observed in the honeybush (*Cyclopia sp*, *Fabaceae*) and *Anemarrhena asphodeloides* (*Liliaceae*) [8–11]. It has been proven that mangiferin exhibits many pharmacological and biological activities. One of these properties is cytotoxic activity against different tumor cells [12]. Mangiferin was found to induce apoptosis in the human acute myeloid leukemia cell line HL-60 [13]. In our previous study, we identified that mangiferin enhanced apoptotic effects of hesperidin in the human cervix adenocarcinoma cell line (HeLa) [14]. On the other hand, dermal acute studies of mangiferin in rodents did not show toxic effects of this compound in mice and rats after exposure to 2 g/kg mangiferin doses [15].

An important pharmacological activity of mangiferin is its strong antioxidant properties [16, 17]. Thus, many studies have been focused on the preparation and implementation of mangiferin formulation on the skin. However, there are only a few studies showing the solubility of mangiferin in the solvents used in the cosmetic and pharmaceutical industry. It is known that mangiferin is slightly soluble in ethanol, sparingly soluble in water and practically insoluble in some nonpolar solvents (e.g. n-hexane, diethyl ether) [18].

The mangiferin permeation into the skin and through the skin, as an influence on the skin’s condition, have not been analyzed so far. In the xenobiotic passage process to the skin and through the skin, the stratum corneum is the most important barrier. It has been found that the greatest ability to overcome the stratum corneum is related to compounds whose log P is between 1 and 3, a molecular weight of less than 500 Da [19]. Although mangiferin is an aglycoside possessing branched structure [20], its molar mass is 422.33 g/mol and the log P is 2.73 [21], which suggests that mangiferin could be able to pass through the stratum corneum and interact with ECM enzymes in deeper, living skin layers.

The aim of our study was to analyze the penetration of mangiferin into the human skin and through the skin. The influence of mangiferin on two Extracellular Matrix Enzymes (ECM): collagenase and elastase, was also evaluated.

## Materials and methods

### Materials

Collagenase from *Clostridium histolyticum*, neutrophil elastase, tricine buffer, N-[3-(2-Furyl)acryloyl]-Leu-Gly-Pro-Ala (FALGPA), N-Succinyl-Ala-Ala-Ala-p-nitroanilide (SANA), mangiferin, oleanolic acid were sourced from Sigma Chemical Co. (St. Louis, MO, USA), and TRIS-HCl, NaCl, CaCl_2_, DMSO were sourced from Avantor Performance Materials Poland S.A.

### Permeation study

The studies were performed using human cadaver full skin (thickness of 0.75 mm) obtained from the region of the thorax of 40–60 years old donors. The study was reviewed and approved by the Independent Bioethics Commission for Research of the Medical University of Gdansk (number NKBBN/120-41/2014). Before the experiment, the skin was stored frozen at -20^°^C. The preparations were applied as an infinite dose (0.5 ml mangiferin solution at concentration of 25 μg/ml) on the surface of the skin placed in the flow-through Teflon^®^ diffusion chamber (Crown Glass, USA), and left for 24 h. The diffusion area of the skin was 0.65 cm^2^. The donor compartment of the chamber remained occluded, and the system was maintained at the temperature 37°C ± 0.5°C. Saline solution, 20 ml, preserved with 0.005% sodium azide (Fluka, Buchs, Switzerland) was recirculated beneath the skin with a constant rate of 10 ml/h. Acceptor fluid ensured the sink condition. After the penetrants were removed from the skin, the stratum corneum was separated by tape-stripping method, using 30 fragments of an adhesive tape (3M Medica Pharma, St. Paul, MN, USA). The following parameters were employed: pressure about 1 kg/cm^2^ (applied by stamp), 2 s duration of pressure, and a rapid removal rate at an angle of 45^o^. The skin layers were isolated by the heat separation technique [22]. The whole skin was immersed in water at 60°C for 45 s, followed by careful removal of the epidermis by tweezers. All skin layers were extracted with 5.0 ml of methanol (HPLC-grade, P.O.Ch., Gliwice, Poland).

### Fluorescence microscopy

According to the fact that mangiferin is able to give fluorescence, an additional experiment was done. The aim of it was to confirm the distribution of mangiferin in the different skin layers with the use of fluorescence microscopy. The maximum excitation of mangiferin and emission were 248 nm and 520 nm, respectively [23].

The experiment was performed analogously as in the diffusion study. The ethanol solution of mangiferin applied on the skin was 75 μg/ml (96% EtOH), whilst in the control study only ethanol was applied. During the analyses, samples from the receiver solution were not collected. After 24 hours the skin was removed from the diffusion cells without separation and frozen at -60°C in Cryotome E (Thermo Electron Corporation). Afterwards, several cross-cuttings of the skin were performed in order to obtain the transverse profile of the skin. Shredded boundaries were discarded. Cuttings, which had all layers of the skin transverse profile visible, were placed on a laboratory slide. Immediately after thawing skin samples were analyzed with a fluorescence microscope Nikon Eclipse 50, filter UV 2A: 330 nm excitation, 380 nm emission, equipped with the lamp–Nikon super high pressure mercury–and the camera Nikon 05–5MC. The acquired images (magnification 100 times) were analyzed in the NIS Elements AR3.2 program.

### Chromatography

The HPLC system consisted of a spectrophotometric diode array 340S detector pump P 580, an automated sample injector ASI-100, and column thermostate STH 585, all from Dionex Corporation (Sunnyvale, CA, USA) and the Coulochem II electrochemical detector–equipped with a 5020 model guard and a 5010 model analytical cell (ESA, Chelmsford, MA, USA) operated by the Chromeleon chromatography-management system (version 6.8 (Dionex)). Compounds were separated on a Hypersil Gold C_18_ column (150 mm × 4.6 mm I.D., 5 μm particle) with the Hypersil Gold guard column (10 mm × 4.6 mm I.D., 5 μm particle), both from Thermo Electron Corporation (Dreieich, Germany).

The isocratic mobile phase was 15 mM sodium phosphate, pH 4.0 with 85% orthophosphoric acid and acetonitrile (65/35 v/v). The flow rate was 1.0 ml min^−1^. The mobile phase was filtered through a 0.22-μm membrane filter and vacuum degassed before use. The injection volume was 20 μl. The column and automated sample injector thermostats were set at 20 and 8°C, respectively. The electrochemical behavior of mangiferin was studied by the repeated injection of working standard solutions (0.1 mg/ml) and by detection at potentials from -0.5 to +1.2 V. Hydrodynamic voltammograms of the analytes exhibited good responses in the ranges from +0.35 to +0.95 V. The potentials applied were: the guard cell +1.1 V, first working electrode (E1) +0.35 V, and second working electrode (E2) +0.95 V. Detection was confirmed by a photodiode array detector at 225, 254, 280 and 360 nm wavelengths, respectively [24].

### Enzyme assay

#### Elastase assay

A spectrophotometric elastase assay was performed as previously described [25], with *N*-Succ-(-Ala)_3_-*p*-nitroanilide (SANA) as the substrate. Each reaction mixture contained Tris-HCl buffer (pH 8), 1.0 μg/mL porcine pancreatic elastase, 0.8 mM SANA and different concentrations of mangiferin (0–600 μM dissolved in 25% DMSO). Reaction mixtures were pre-incubated with mangiferin solutions for 15 minutes at room temperature. The addition of SANA started the reaction. Changes in product formation: p-nitroaniline was recorded every 20 seconds for 20 minutes in the absorbance λ = 410 nm with the use of the Epoch (BioTek System) spectrophotometer in 96-well plates (BIOCOM SYSTEMS). The oleanolic acid was used as a control [26]. Kinetic parameters Vo, Km and Vmax were calculated according to Michaelis-Menten and Lineweaver-Burk plots and analyzed in the GraphPad prism program and Excel.

As stated by the data supplier: 1U elastase hydrolyses 1 μM SANA/min at pH 8 and 25°C. The molar absorption coefficient of p-nitroaniline is 9800 M^–1^ cm^–1^.

#### Collagenase assay

A spectrophotometric collagenase assay was performed as previously described [25], with N-[3- (2-Furyl) acryloyl]- Leu- Gly- Pro-Ala (FALGPA) as the substrate. Each reaction mixture contained a 50 mM tricine buffer (pH 7.5 with 400 mM NaCl and 10 mM CaCl_2_), 0.8 mM FALGPA, 0.1 units of collagenase and different concentrations of mangiferin (0 μM—650 μM dissolved in 25% DMSO). The reaction mixtures were preincubated with mangiferin solutions for 15 minutes at room temperature. The addition of FALGPA started the reaction. Changes in product formation were recorded every 20 seconds for 20 minutes in the absorbance λ = 335 nm with the use of the Epoch (BioTek System) spectrophotometer in 96-well plates (BIOCOM SYSTEMS). Oleanolic acid was used as a control. The kinetic parameters Vo, Km and Vmax were calculated according to Michaelis-Menten and Lineweaver-Burk plots and analyzed in the GraphPad prism program and Excel.

As stated by the data supplier: 1U of collagenase hydrolyses 1 μM FALGPA/min at pH 7.5 and 25°C, in the presence of calcium ions. The FALGPA molar absorption coefficient is 24700 M^–1^ cm^–1^.

### Statistical analysis

#### HPLC analysis

The calibration curves for mangiferin showed good linearity in the investigated range (10.0–100.0 ng, R^2^>0.9995). The limit of detection (LOD) was 0.35 ng/ml and the limit of quantification (LOQ) was 9.7 ng/ml. Precision and reproducibility were evaluated by six replicated analyses, and the R.S.D. values were less than 0.9% and 3.7%.

#### Skin permeability

All data are expressed as mean values ± standard deviation (SD). Statistical comparisons of the results were evaluated using a two-way ANOVA with post-hoc Tukey test (p<0.05).

#### Enzyme assay

The results are shown ± standard deviation (SD). Statistical significance between samples was determined using the Student's t-test (n = 8, p<0.05). Each analysis was conducted in two independent analyses, with five repetitions in each. The data were analyzed using the GraphPad Prism, and Excel.

## Results

The obtained results indicate that mangiferin is able to cross stratum corneum and permeate into the deeper skin layers: the dermis and epidermis. Beside the accumulation in the skin, mangiferin penetrates through the skin and is presented in the acceptor fluid.

In order to quantify the penetration of mangiferin into the skin and through the skin, HPLC analysis with electrochemical detection was used.

The fluorescence microscopy has demonstrated extracellular distribution of mangiferin after application on the skin directly from the thin slices of skin. The method does not require either a long time or additional analysis.

In the skin, collagenase and elastase occur in the epidermis and dermis where mangiferin is able to penetrate and inhibit their activity.

### Accumulation of mangiferin in the skin layers

**Fig 2:**
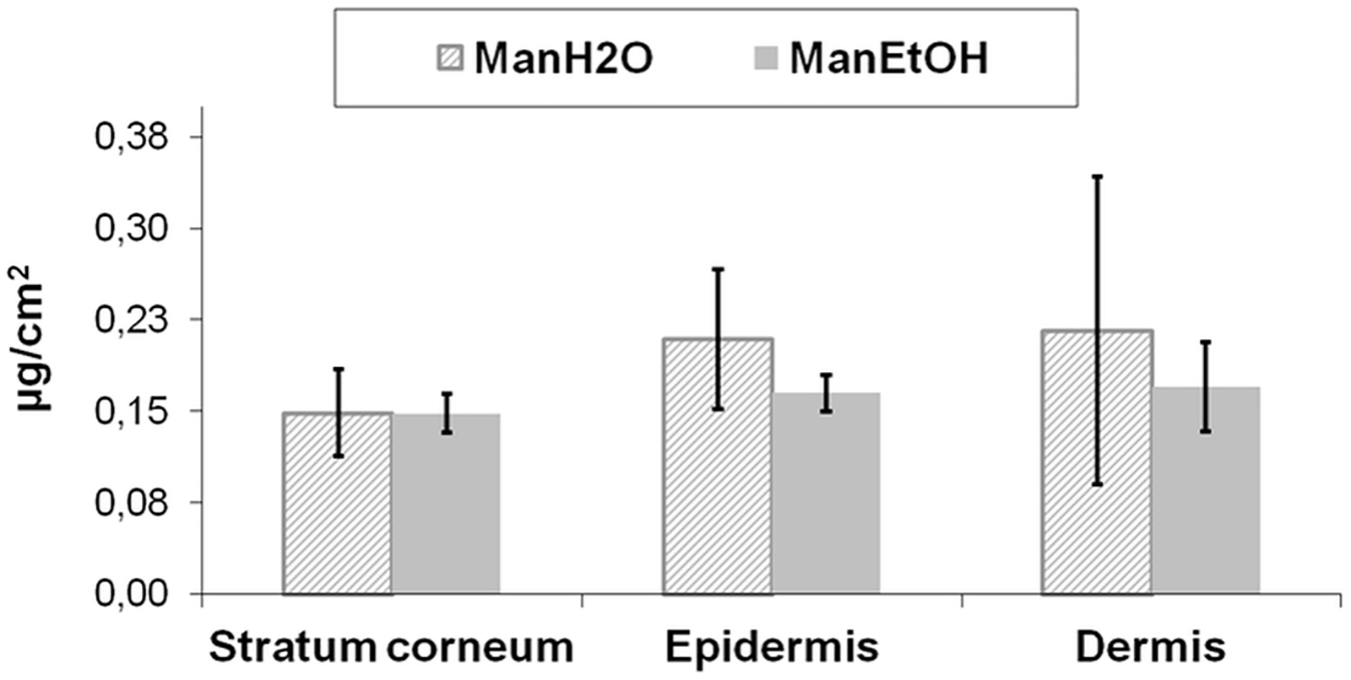
Absorption of mangiferin applied as aqueous ManH_2_O and ethanol ManEtOH solutions in the skin layers: the stratum corneum, epidermis and dermis (μg/cm^2^). After 24 h of mangiferin application, the quantitative content of the compound in all the skin layers was measured with HPLC. The obtained HPLC data (ng/ml) were used to calculate the amount of mangiferin that permeates to the skin surface area of 1 cm^2^. Error bars represent standard deviations. Statistical analysis did not show significant differences among the groups (p>0.05, two-way ANOVA with post-hoc Tukey test).

Mangiferin’s ability to permeate through the human skin was verified during the “ex vivo” analysis. It has been confirmed with the use of HPLC equipped with electrochemical detection that after 24 h analysis mangiferin was present in all the skin layers (Fig 2). The least amount of mangiferin was presented in the stratum corneum (equal amounts despite solution type), whilst the mangiferin concentration differences between the epidermis and dermis were insignificant. It has also been shown that mangiferin applied from aqueous solution presents a higher accumulation in the epidermis and dermis in comparison with ethanol solution.

### Permeation of mangiferin through the skin

**Fig 3:**
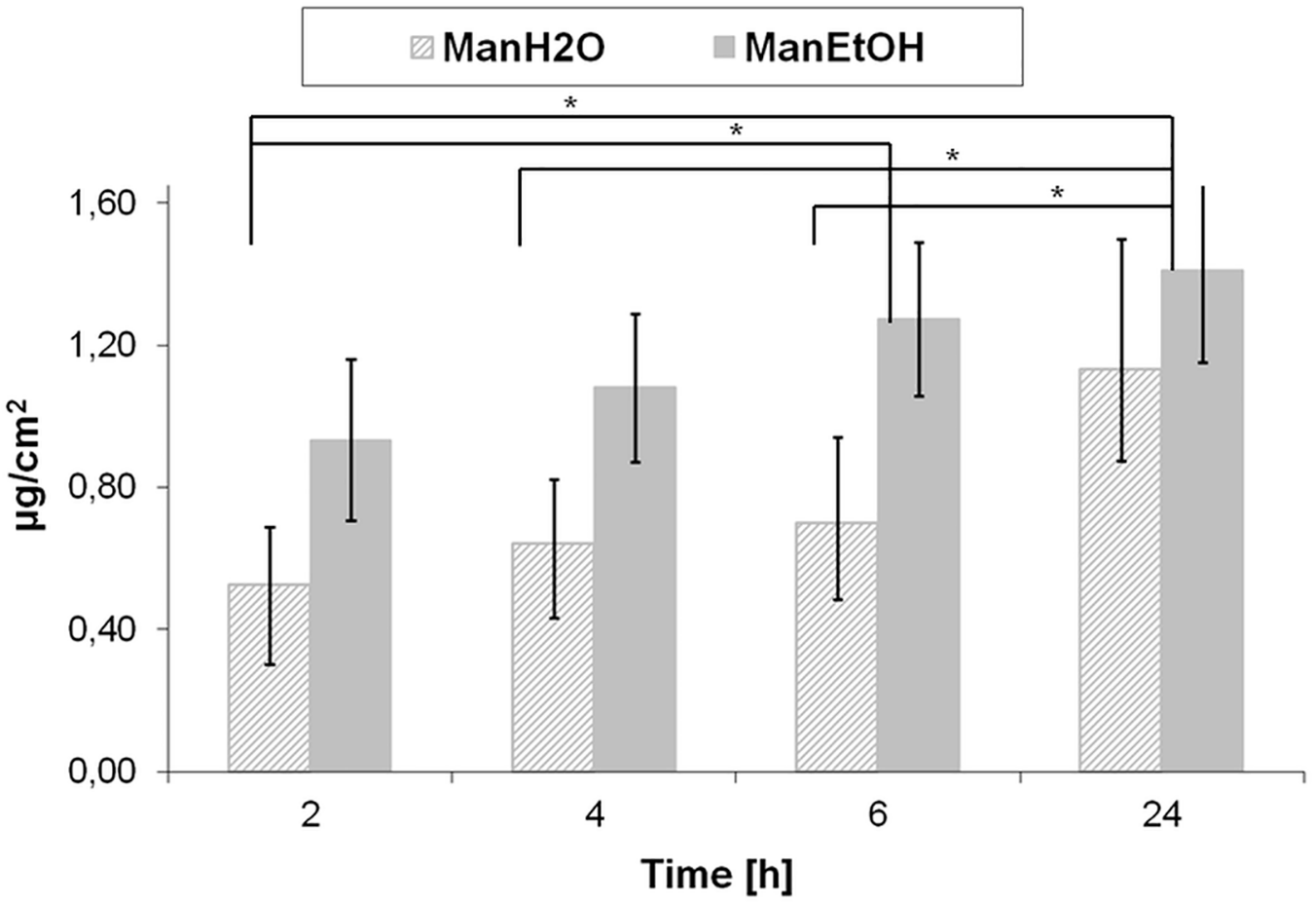
Permeation of mangiferin through the skin. The aqueous and ethanol mangiferin solutions were applied on the skin and after 2, 4, 6 and 24 h the amount of mangiferin in the acceptor fluid was measured by HPLC. The obtained HPLC data (ng/ml) were used to calculate the amount of mangiferin that permeates through the skin surface area of 1 cm^2^. Error bars represent standard deviations. Significant differences between the results are marked with an “*” (p<0.05, two-way ANOVA with post-hoc Tukey test).

During the 24 hours analysis, four samples were taken from the acceptor fluid (after 2, 4, 6 and 24 h) (Fig 3). In all the analyzed probes mangiferin was detected. The data indicate that the highest mangiferin amount that permeated through the skin was detected within the first 2 hours. After the next few hours the mangiferin concentration in the acceptor fluid was increased continuously but not in a linear manner. After 24 hours the mangiferin concentration in the acceptor fluid was about 2-fold higher than after 2 hours. It has also been shown that mangiferin dissolved in ethanol presents a higher permeation through the skin than the water mangiferin solution. The results also indicated that during the first 6-hour analysis the amount of mangiferin from ethanol solution that permeated through the skin was about 2-fold higher than from the water solution.

### Distribution of mangiferin in the skin

Assuming that 100% of the mangiferin is able to penetrate or permeate the skin, it is possible to analyze the mangiferin percentage distribution in the individual layers of the skin and the acceptor fluid. In our study, we analyzed the mangiferin amount that had absorbed in all the skin layers (Fig 2), and permeated through the skin (Fig 3).

**Fig 4:**
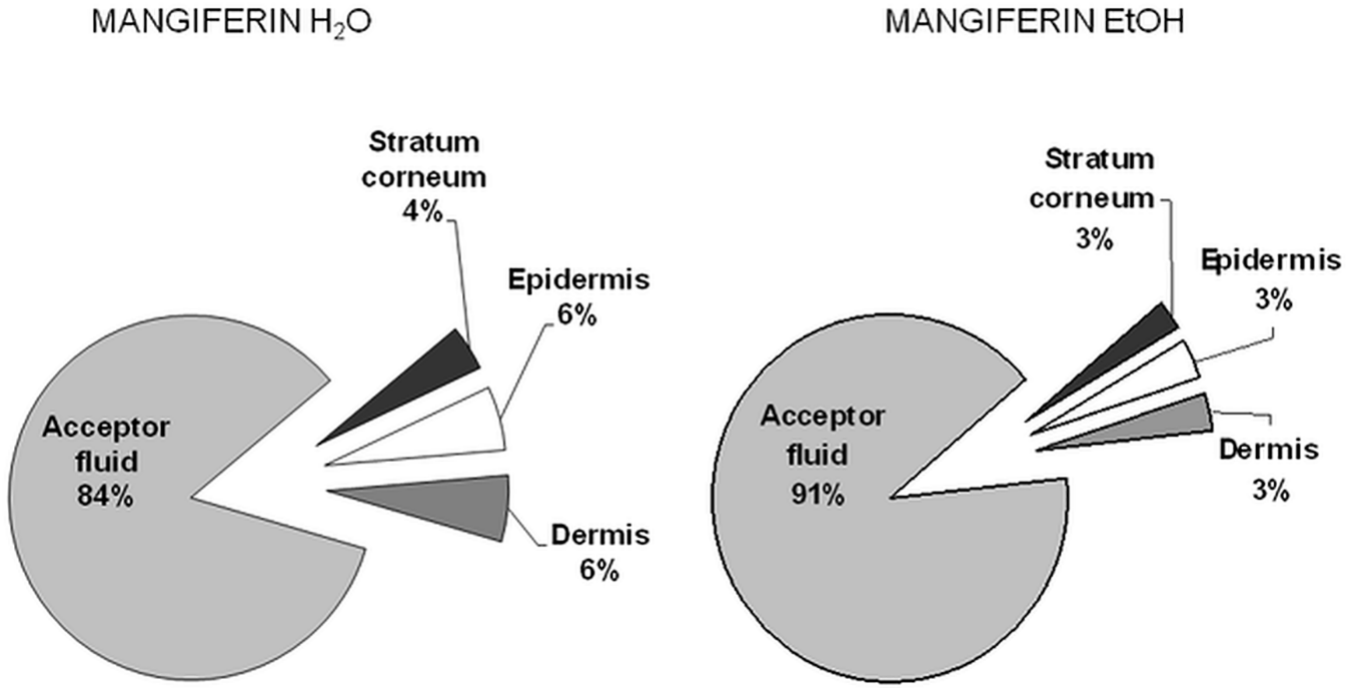
Distribution of mangiferin in the individual layers of the skin and the fluid acceptor.

After 24 h of mangiferin application, 16% and 9% of mangiferin was accumulated in the skin from the aqueous and ethanol solutions, respectively. In the acceptor fluid there was 84% and 91% of mangiferin coming from the aqueous and ethanol solutions, respectively (Fig 4).

Due to the fact that mangiferin is a substance having condensed heterocyclic rings, which determines its ability to fluoresce [23], an additional experiment with a fluorescent microscope was performed to analyze the dermal mangiferin distribution in the various layers of the skin. The fluorescence microscopy is used to visualize the distribution of natural compounds absorbed by the skin [27]. The intensity of fluorescence in the individual layers of the skin provides preliminary information on the distribution of the xenobiotic across the skin, without the need for a long lasting and expensive pilot quantitative analysis. Some interaction between the chemical that is able to take an electron and skin macromolecules can also lead to the loss in fluorescence intensity, called *quenching* [28].

**Fig 5:**
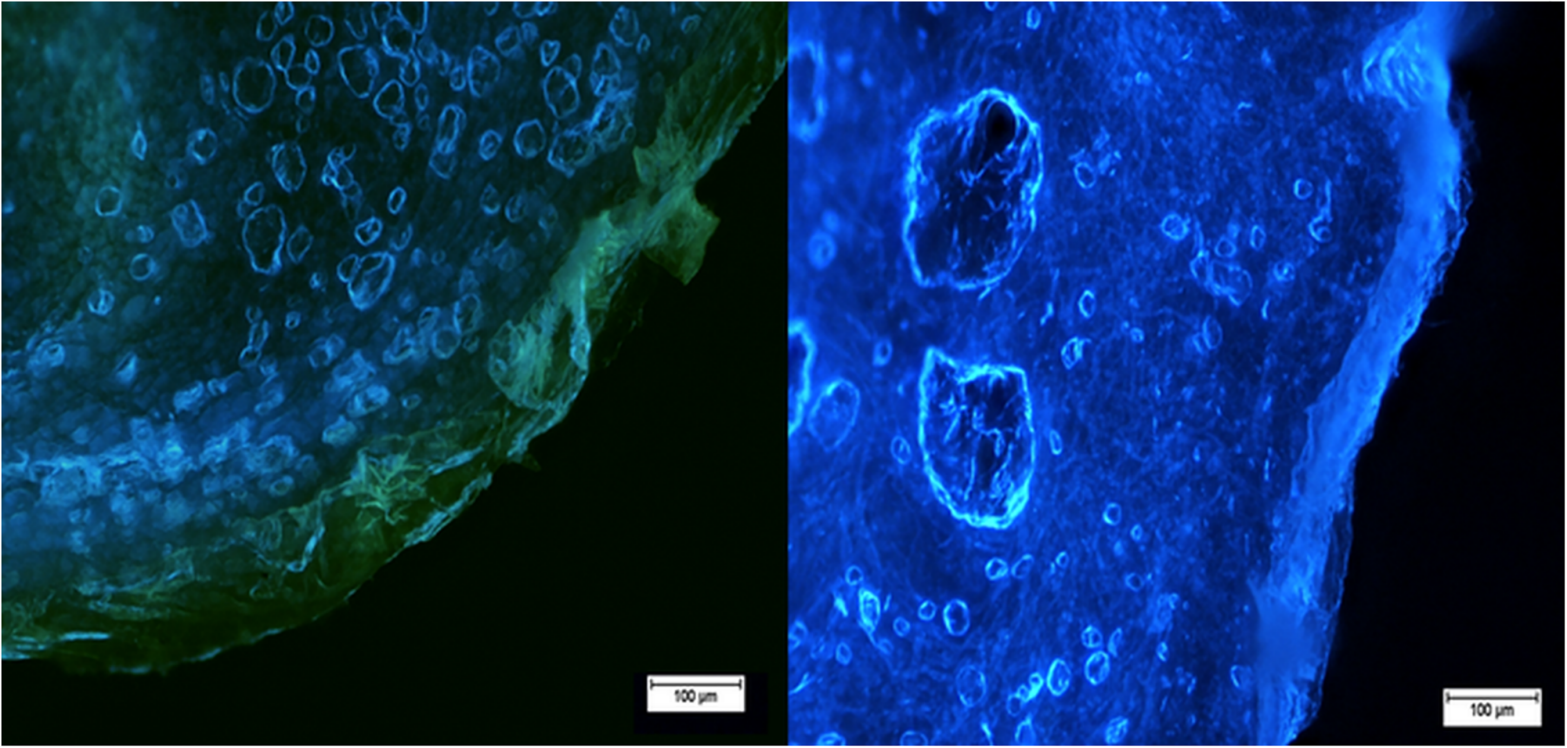
Penetration of mangiferin in the human skin. The ethanol (96%) as a control (right image) and ethanolic solution of mangiferin (75 μg/ml) (left image) were applied on the skin. After 24h, the skin was observed under the fluorescence microscope Nikon Eclipse 50, filter UV 2A, Ex 330–380 nm.

Observation of mangiferin distribution in the individual layers of the skin was possible as mangiferin exhibits fluorescence at different wavelengths than the macromolecules of the skin. The right image (Fig 5) presents fluorescence of the skin on which only ethanol was applied, and the left skin image presents fluorescence caused by the application of the ethanolic solution of mangiferin (75 μg/ml, 96% EtOH).

The results presented in Fig 5 demonstrate a visualization of the more or less even distribution of mangiferin in the living layers of the skin (according to the HPLC analysis). The permeation of mangiferin to the dead cells of the stratum corneum causes a strong yellow-green fluorescence characteristic for the applied xanthone [23].

**Fig 6:**
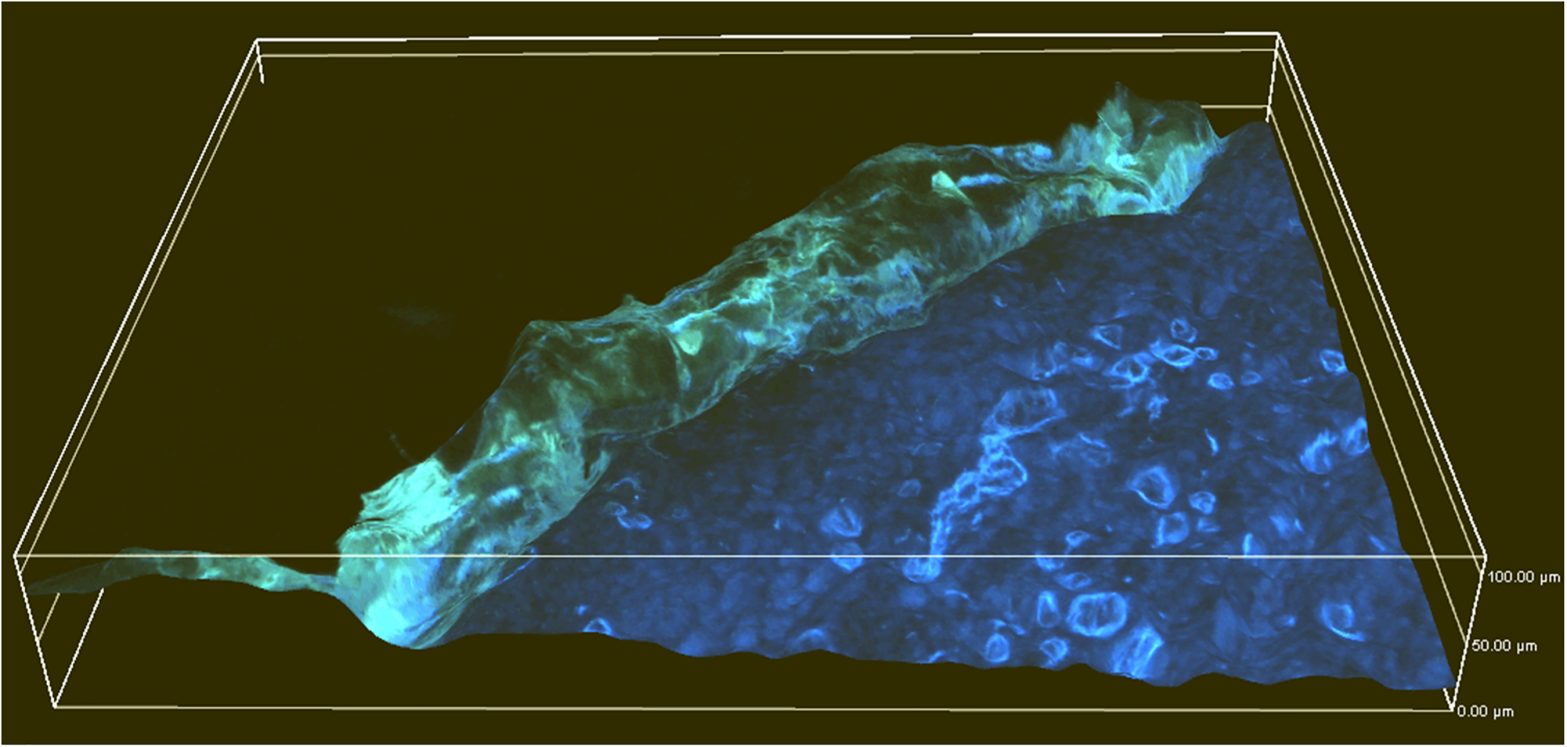
Three-dimensional image of mangiferin (75 μg/ml in 50% EtOH) absorption in the skin. Image was formed by overlapping multiple images of the same area of skin at various focal planes (NIS Elements AR3.2 program).

Of particular note is the observation of the reduced autofluorescence intensity of the epidermis and dermis after application of the ethanol mangiferin solution. As the photograph in Fig 6 shows (the three-dimensional image), a significant loss of fluorescence in the epidermis and dermis and a yellow-green fluorescence of the stratum corneum were observed. Silencing of the skin’s autofluorescence may be caused by the interaction of mangiferin with active molecules in the living skin layers.

### Effect of mangiferin on elastase activity

Mangiferin was investigated for its anti-elastase activity using SANA as the substrate. The type of inhibition, and the reversibility of the inhibition process, were analyzed. Samples with different mangiferin concentrations were prepared: 0–600μM.

**Fig 7:**
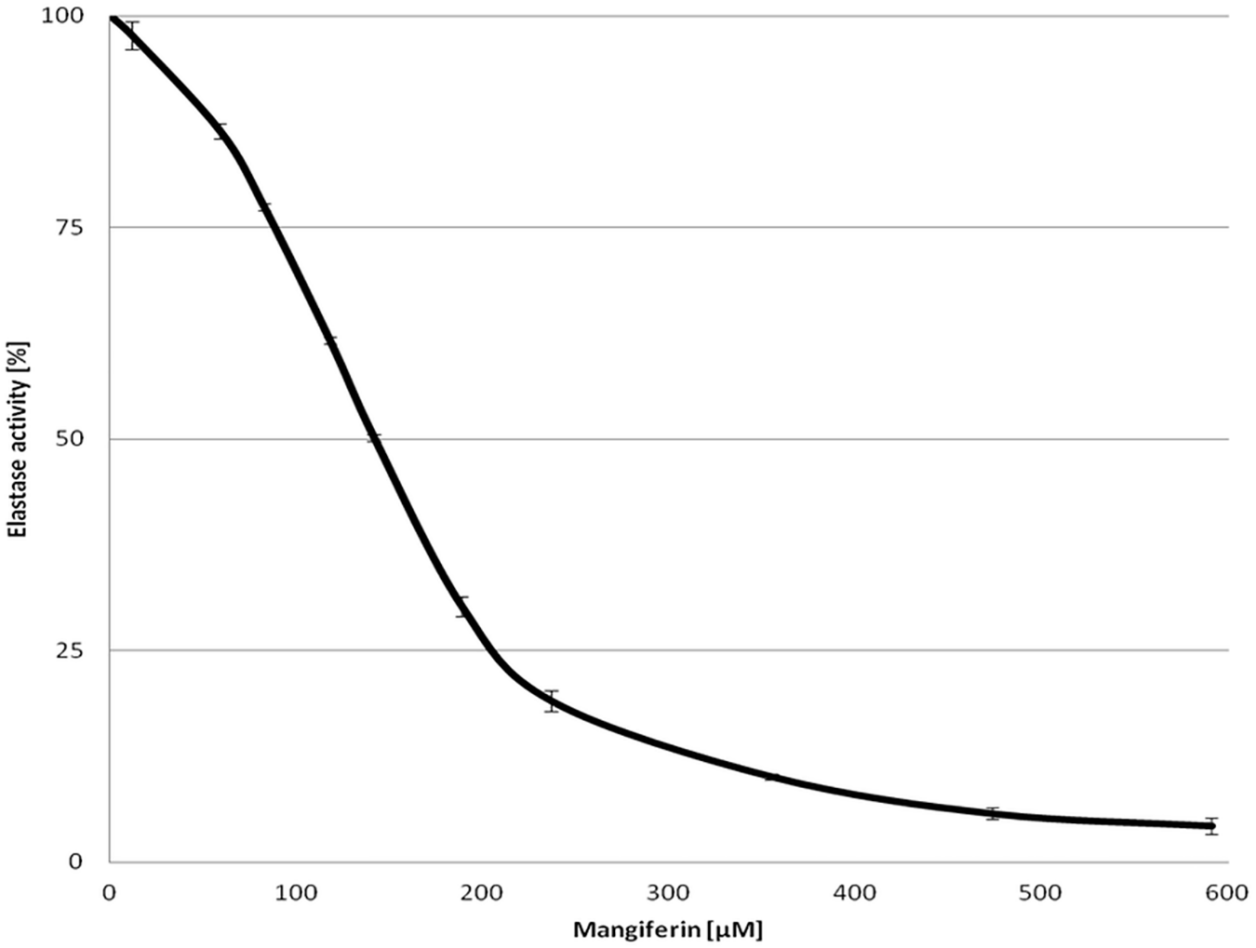
Changes in the activity of elastase (%) in the presence of increasing concentrations of mangiferin solution (μM).

Elastase activity was inactivated by mangiferin in a dose-dependent manner. The mangiferin concentration leading to a 50% loss of elastase activity (IC50) was 139.64 ± 9.34 µM. IC50 of oleanolic acid was 33.39 ± 2.25 μM. Mangiferin concentrations above 473 μM inactivated elastase almost completely (Fig 7).

**Fig 8:**
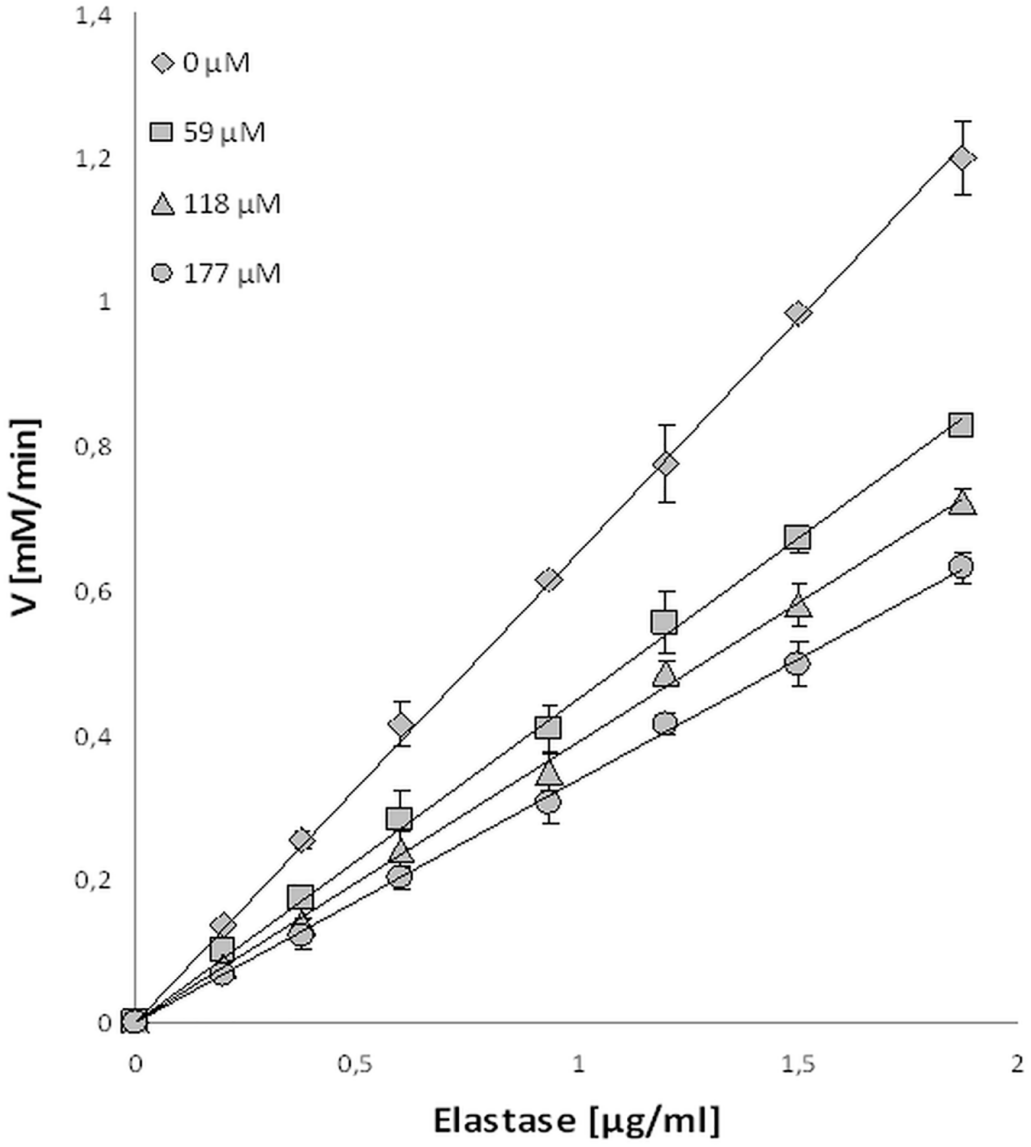
Activity of elastase (mM/min) vs elastase concentration (μg/ml) in the presence of different concentrations of mangiferin solution (0, 59, 118 and 177 μM).

In order to confirm the reversibility interaction between mangiferin and elastase, plots of the enzyme’s activity to elastase concentration in different mangiferin concentration were drawn (Fig 8). The analyses have shown straight lines passing through the origin. As the inhibitor concentration increased, the slope of the lines decreased. This kind of relationship between enzyme activity and its concentration in the presence of the inhibitor indicates the reversibility of the mangiferin inhibition effect on elastase [29].

**Fig 9:**
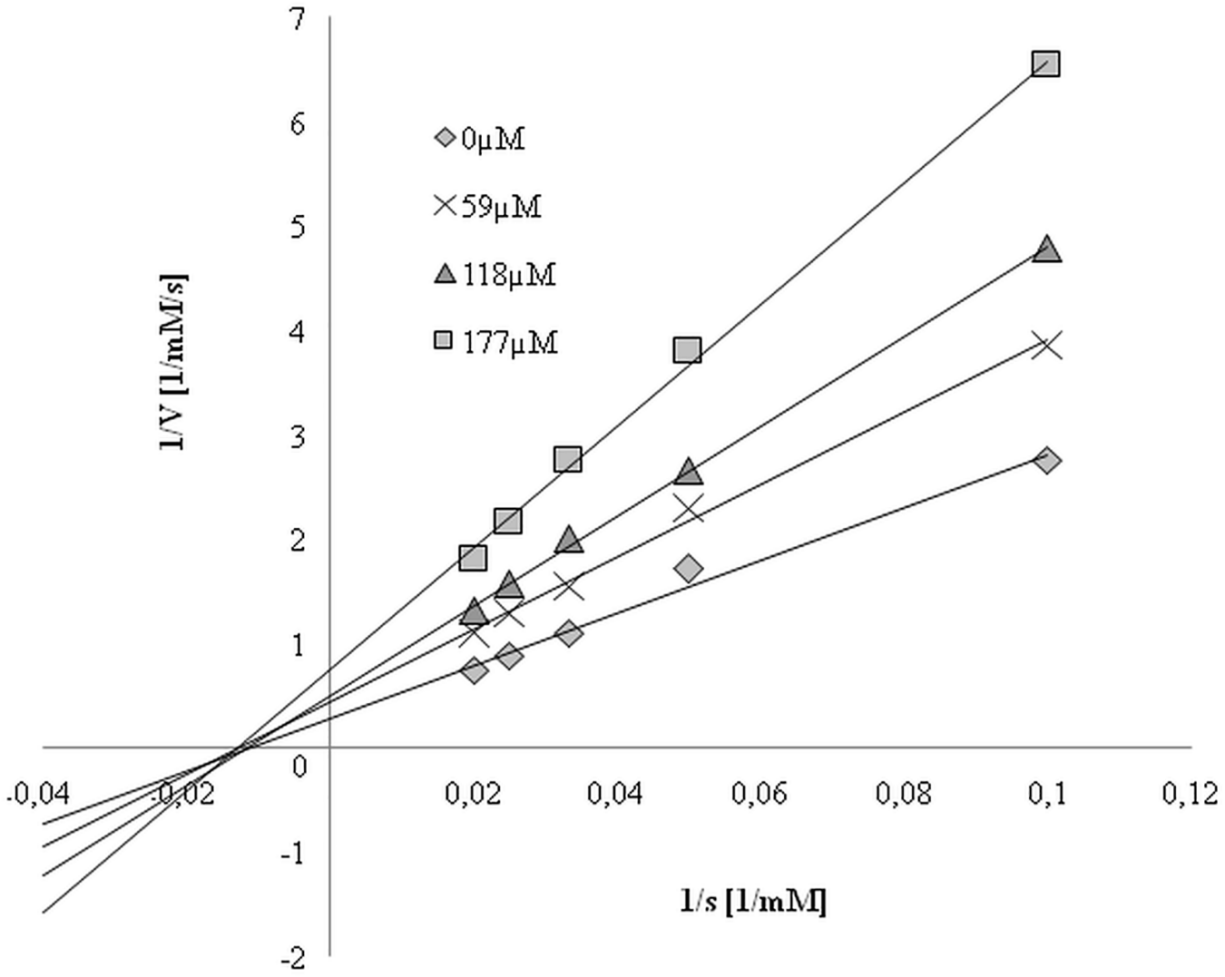
Lineweaver-Burk plots of elastase reaction in the presence of different concentrations of mangiferin solution (0, 59, 118 and 177 μM).

A comparison of the kinetic parameters of the elastase reaction without and with different concentrations of mangiferin was made and the Lineweaver-Burk plots were analyzed. Fig 9 shows changes in the Vmax value with the increasing inhibition concentration, while the Km revealed no changes in the same reaction conditions. Results indicate a noncompetitive type of mangiferin inhibition on elastase: mangiferin shows an affinity both for the free enzyme as well as the enzyme-substrate complex.

### Effect of mangiferin on collagenase activity

Mangiferin was investigated for its anti-collagenase activity using FALGPA as the substrate. The type of inhibition and reversibility of the inhibition process were analyzed. Samples were prepared with different mangiferin concentrations: 0–650 μM.

**Fig 10:**
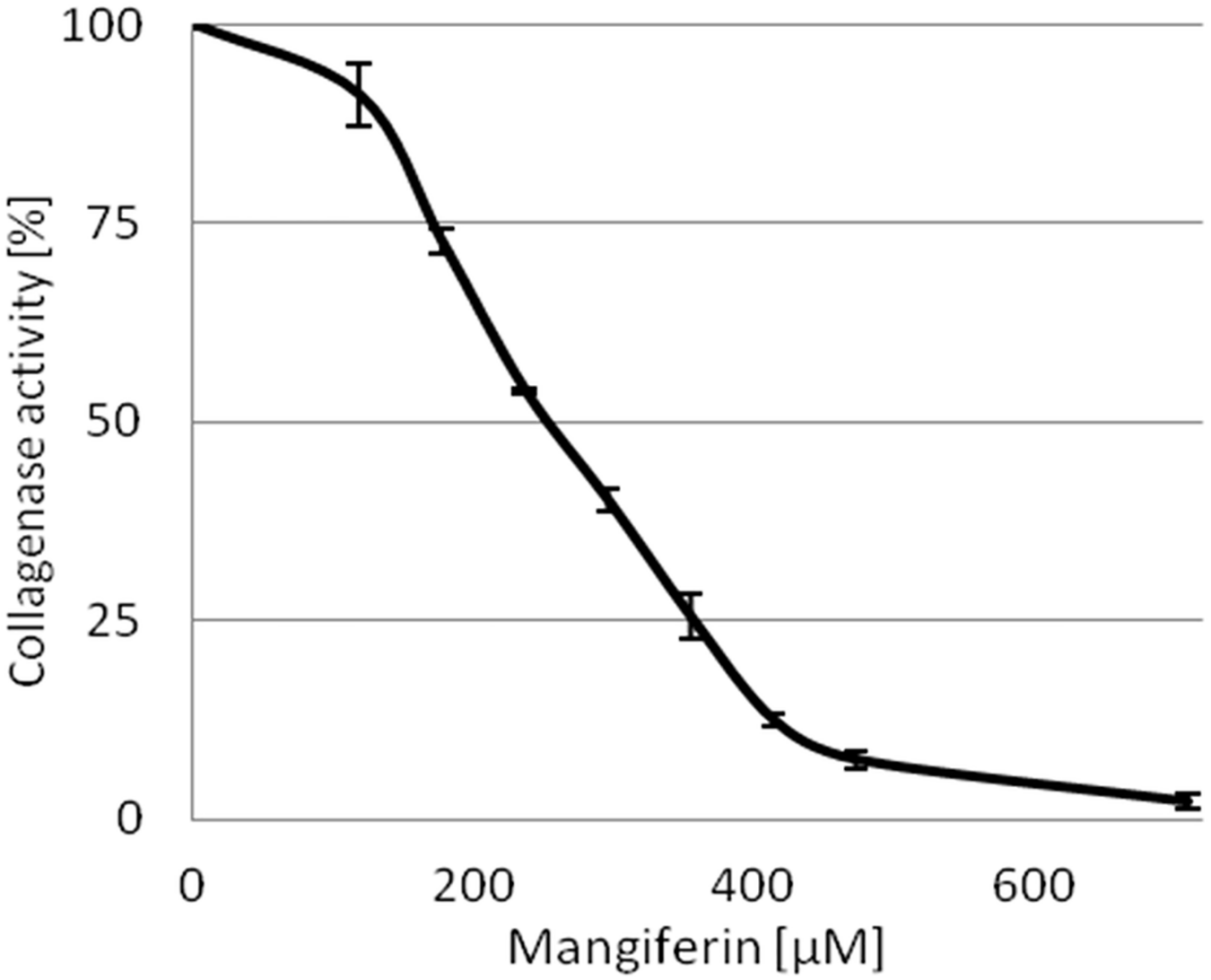
Changes in the activity of collagenase (%) in the presence of increasing concentrations of mangiferin solution (μM).

Collagenase activity was inactivated by mangiferin in a dose-dependent manner. The mangiferin concentration leading to a 50% loss of collagenase activity (IC50) was 253.57 ± 7.56 μM. Mangiferin concentrations above 500 μM inactivated collagenase almost completely (Fig 10). The IC50 of oleanolic acid was 51.75 ± 5.25 μM.

**Fig 11:**
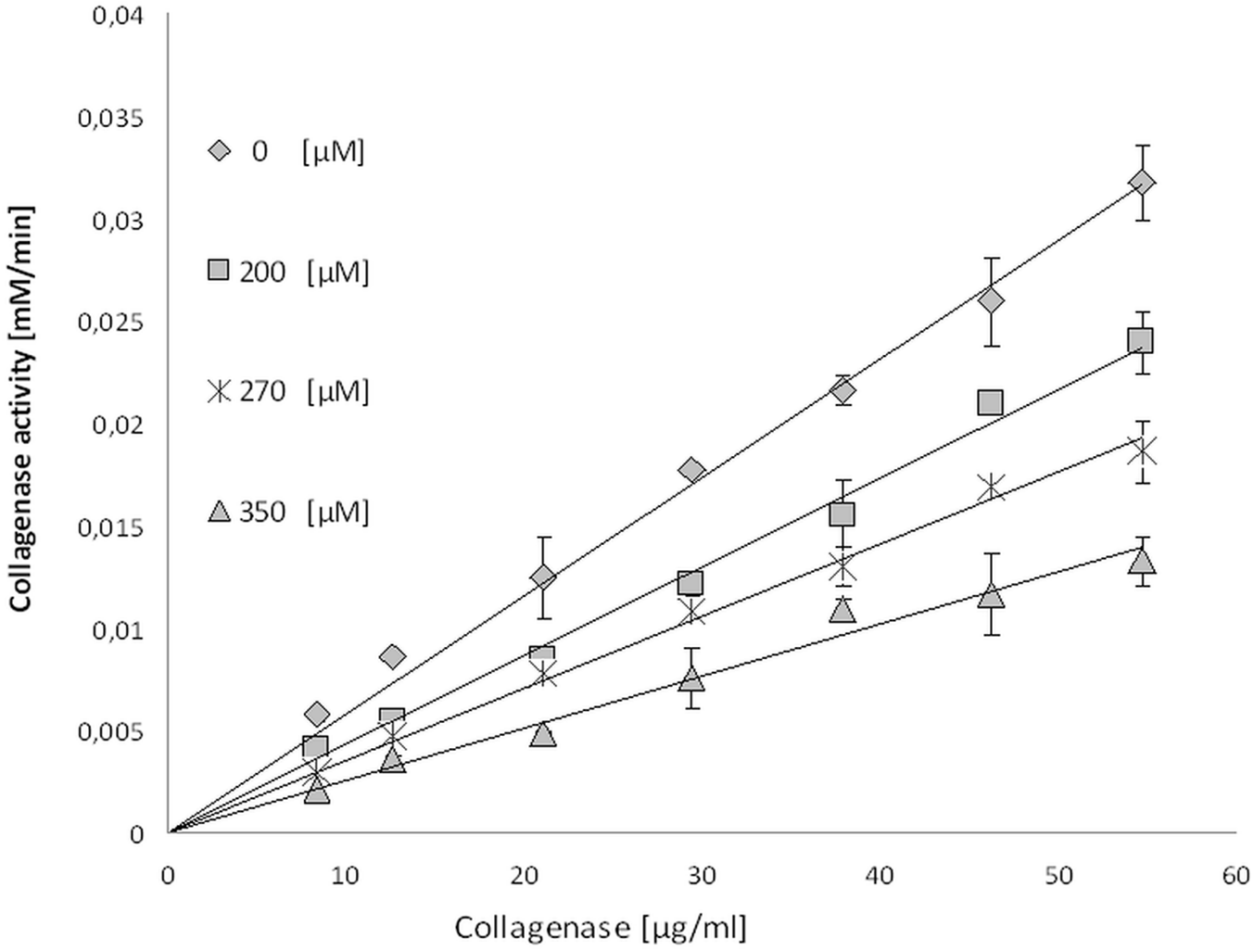
Activity of collagenase (mM/min) vs collagenase concentration (μg/ml) in the presence of different concentrations of mangiferin solution (0, 200, 270 and 350 μM).

In order to confirm the reversibility interaction between mangiferin and collagenase, plots of the enzyme activity in relation to collagenase concentration in different mangiferin concentrations were drawn (Fig 11). The analyses have shown straight lines passing through the origin. As the inhibitor concentration increased, the slope of the lines decreased. Thus, the results indicate that mangiferin inhibits collagenase activity in a reversible manner.

**Fig 12:**
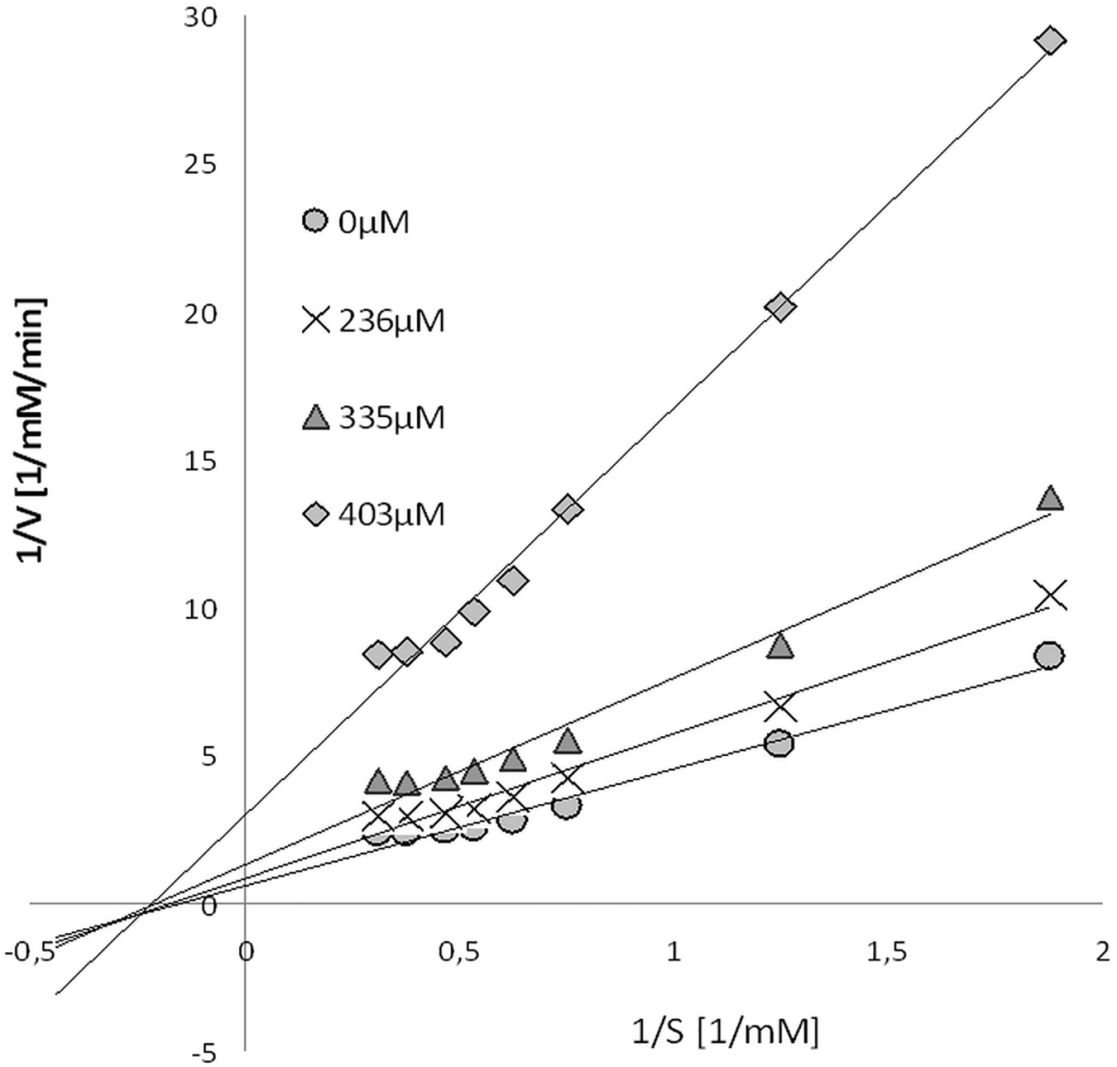
Lineweaver-Burk plots of collagenase reaction in the presence of different concentrations of mangiferin solution (0, 236, 335 and 403 μM).

A comparison of the kinetic parameters of the collagenase reaction without and with different concentrations of mangiferin was made and the Lineweaver- Burk plots were analyzed. Fig 12 shows changes in the Vmax value with the increasing inhibition concentration, while the Km revealed no changes in the same reaction conditions. Results indicate a noncompetitive type of mangiferin inhibition on the collagenase: mangiferin shows an affinity both for the free enzyme, as well as the enzyme-substrate complex.

## Discussion

Mangiferin, a branched structure glycoside, demonstrated the ability to penetrate into the skin and also to pass through the skin (human skin, *ex vivo* study). The stratum corneum barrier did not stop the mangiferin. It might be associated with the fact that the mangiferin log P is between 1 and 3 (mangiferin log P = 2.73), corresponding to a weight which is less than 500 Da (422.34 Da) [19, 21]. To confirm the quantitative distribution of analyzed compound, HPLC equipped with electrochemical detection was used. The compound penetrated through the stratum corneum without significant differences in accumulation between the skin layers (for the water solution and for the ethanol solution respectively: stratum corneum- 4% and 3%, epidermis- 6% and 3% and dermis: 6% and 3%). The most considerable amount of mangiferin was observed in the acceptor fluid (84% for the water solution, 91% for the ethanol solution), which suggests a greater capability of mangiferin to pass through the skin than to accumulate into the skin. The studies have demonstrated the superiority of mangiferin permeability through the skin layers as compared to its accumulation in the skin.

The use of fluorescent microscopy was sufficient to visualize the mangiferin distribution in the skin layers without the need for long lasting quantitative analysis and the separation of layers. The utility of the method is particularly valuable in the case of compounds that are able to give fluorescence in a different wavelength range than skin autofluorescence [30].

It has been shown that mangiferin has the ability to quench the autofluorescence of the living skin parts (epidermis and dermis). Quantitative analysis indicated a comparable accumulation of mangiferin in the epidermis and dermis, and the living layers after absorption showed remarkable calm fluorescence (called quenching). This demonstrates the ability of mangiferin to donate electrons and that it interacts with macromolecules. The literature data confirm that mangiferin is able to form complexes with human serum albumin (HAS) [31], and bovine serum albumin (BSA) [32]. This may have a significant impact on mangiferin application or in the designing of new molecules based on its skeleton in order to block the expression of specific genes or the function of proteins and enzymes. The validity of the application confirms the fact that, as shown in this study, mangiferin creates interactions with macromolecules of the skin and enzymes (collagenase and elastase).

The use of fluorescence microscopy in order to analyze changes in endothelium has gained popularity in the recent years [33]. In particular, great expectations are associated with the phenomenon of the suppression of the autofluorescence of tissues and organs by the compounds having a moiety of fluorophore, and which are therefore capable of transferring electrons. The resulting complexes may be indicative of changes in the conformation of macromolecules [28]. Because of this effect it is possible to observe fluorophore distribution, for example, in the skin and cancerous tissue. PDT (Photodynamic Therapy), a non-invasive method (no scalpel used) to destroy the tumor, is based on providing to the tissue an anticancer substance (photosensitizer) which is selectively retained by tumor cells. When the tumor is irradiated by light, the anticancer substance is activated leading to the reactive oxygen species (ROS) generation and destruction of the cancer cells [34]. Recent studies on the application of PDT show the potential use of naturally occurring compounds in this therapy [35]. Thus, mangiferin as a pro-apoptotic agent can be a potentially photodynamic therapeutic candidate in PDT, even in co-administration with different chemotherapeutic agents [12].

The mangiferin has the ability to penetrate through the stratum corneum barrier and to the living skin layers, where collagenase and elastase occur. The *in vitro* analysis revealed that mangiferin is capable of a dose-dependent inhibition of neutrophil elastase and collagenase activity. The IC50 of mangiferin for elastase and collagenase were 139.64 ± 9.34 μM and 253.57 ± 7.56 μM respectively. Although the potency of the analyzed xanthone on collagenase and elastase activity was weaker than the reference substance based on oleanolic acid (4.9-fold and 4.2-fold lower respectively), mangiferin was able to cause almost the complete inhibition of both enzymes. The impact of mangiferin on the inhibition of collagenase activity and collagen synthesis in the skin have been successfully carried out *in vitro* [36], but the mechanism of the reaction, the reversibility, and the mangiferin concentration range that is necessary to affect the inhibition process had not been tested yet. The influence of mangiferin on elastase had never been analyzed before.

As was shown, mangiferin inhibits elastase and collagenase activity in a reversible manner, referred to as a non-competitive inhibition. Mangiferin interacts with both the free form of analyzed enzymes, at a location other than the active site, as well as with the enzyme-substrate complex.

In addition it is highly probable that the inhibition of collagenase and elastase activity would be more significant when conducted *in vivo* due to the fact that mangiferin is a strong antioxidant. Mangiferin fights the ROS generated by e.g. stress or UV exposure that activates the widely understood skin ageing process. In conclusion, the positive mangiferin effect on the skin’s condition and protection might be significant and further study might be worth continuing in the future.

The results of the enzymatic analysis have expanded the knowledge of mangiferin bioactivity. It shows the ability to inhibit the catalytic activity of skin enzymes: elastase and collagenase, responsible for skin ageing. A significant part in reducing the activity of the enzymes may be additionally played by the ability of mangiferin to control free radicals generated by solar radiation. Oxidative stress results in the activation of many ECM enzymes. Therefore, chemical compounds showing photoprotective and antioxidant activity and those which are also able to penetrate the stratum corneum barrier can be important in the fight against skin ageing. One must note that the collagenase and elastase are present in many tissues and organs, where their activity in many instances is associated with pathological conditions.

Summarizing, the effects of mangiferin on the skin indicate that it might be safely and effectively used in cosmetic and dermatological preparations.
